# Feasibility and Acceptability of a Smartphone and Wearable Assessment Protocol for Adolescents with Depression

**DOI:** 10.1007/s10802-026-01464-9

**Published:** 2026-06-18

**Authors:** Jenny Guo, Jennifer Frederick, Lindsey Cunningham, Nicholas C. Jacobson, Aaron J. Fisher, Jeremy W. Pettit, Dana L. McMakin, Mei Yi Ng

**Affiliations:** 1 Department of Psychology and Center for Children and Families, Florida International University, Miami, FL USA; 2 Center for Technology and Behavioral Health, Geisel School of Medicine, Dartmouth College, Lebanon, NH USA; 3 Quantitative Biomedical Sciences Program, Dartmouth College, Hanover, NH USA; 4 Department of Biomedical Data Science, Geisel School of Medicine, Dartmouth College, Lebanon, NH USA; 5 Department of Psychiatry, Geisel School of Medicine, Dartmouth College, Hanover, NH USA; 6 Department of Psychology, University of California, Berkeley, Berkeley, CA USA

**Keywords:** Depressive disorder, Ecological momentary assessment, Actigraphy, Smartphone, Feasibility study, Mobile sensing

## Abstract

Smartphones and wearables are low–burden tools for assessing real–time mood and behavior. Although these methods have been used with adolescents for behavioral tracking (e.g., activity, sleep), less is known about longer–term use (beyond one week) with adolescents with depression and about mobile sensing for monitoring mood for any adolescent population. This study examined acceptability and feasibility of a one–month EMA, actigraphy, and mobile sensing protocol for adolescents with elevated depressive symptoms. Adolescents aged 12 to 18 (*N* = 69; M*age* = 15.46; 67% assigned female at birth; 42% White; 71% Hispanic or Latine; 38% sexual minority) completed EMA surveys on depressive symptoms, processes, and affect multiple times daily via a smartphone app that also collected passive sensor data (e.g., motion, geolocation). An actigraph measured physical activity and sleep. A feedback interview assessed protocol acceptability. Most participants (91%) completed all components, were willing to participate again (91%), and would recommend participation to peers (93%). EMA response rates improved (mean completion 57% to 66%) after shifting to a semi–personalized schedule with extended response windows. Actigraph wear time was high (> 70%) despite device–related issues. Sensor data availability varied by operating system, and privacy concerns influenced participation. Adherence was correlated within and between modalities, suggesting that individual compliance played a central role in consistent engagement. Findings support the feasibility and acceptability of smartphone and wearable methods for capturing real–world mood and behavior in adolescents, however careful attention to design, engagement, and ethical considerations remains essential.

**Supplementary Information:**

The online version contains supplementary material available at 10.1007/s10802-026-01464-9.

## Introduction

Adolescence is a crucial developmental period marked by significant physiological, psychosocial, and emotional changes, along with increased vulnerability to mental health problems such as depression (Dahl, 2004). In the United States, the prevalence of past–year major depressive episodes (MDEs) among adolescents rose from about 2.2 million (9%) in 2004 to over 3.8 million (15.7%) in 2019 (Abuse & Administration, 2020). MDEs are associated with academic and interpersonal difficulties, physical health issues, and elevated risk for suicide (Wilson et al., 2015). Adolescents also experience greater emotional variability and intensity than children or adults, and these dynamics are further disrupted by internalizing problems such as depression (Bailen et al., 2019; Silk et al., 2003). Compared to their typically developing peers, adolescents with elevated depressive symptoms report more intense negative affect, greater variability of positive and negative emotions, and lower ratios of positive to negative affect (Larson et al., 1990; Silk et al., 2011). Capturing these fluctuations in real–world contexts is key to understanding mechanisms underlying psychopathology and informing intervention development.

Smartphones and wearables are well–suited to capturing adolescents’ real–world fluctuations in affect, especially for those who are experiencing depression. In the United States, 95% of adolescents report having access to a smartphone, and most use them frequently throughout the day (Faverio & Sidoti, 2024). Many adolescents are also highly familiar with smartphone technology, making these tools both accessible and intuitive for this age group. These tools offer low–burden, ecologically valid ways to assess adolescents’ emotions and behavior in daily life. Ecological momentary assessment (EMA) captures real–time (or near real–time) subjective thoughts, feelings, and behaviors reported by individuals or close others (e.g., a caregiver or significant other) in natural environments through frequent, repeated sampling (Trull & Ebner-Priemer, 2013). EMA provides fine–grained variations in symptomatology (e.g., moment–to–moment changes) and reduces risk of recall bias when compared to traditional assessments that rely on retrospective symptom ratings at discrete time points—an important consideration for youth with depression, who tend to have higher recall of negative affect and experiences (Bone et al., 2021). Actigraphy and mobile sensing both collect passive data (requiring minimal additional effort from the user) that have been used in algorithms to identify physiological and behavioral indicators of depression. Actigraphs use accelerometers to detect physical movements on the wrist, waist, or ankle, to estimate sleep duration and efficiency, as well as episodes and levels of physical activity. Smartphones have a suite of sensors including accelerometers, but also global positioning system (GPS) to detect reduced physical activity, call or text frequency logs to detect social withdrawal, content analysis of text logs to detect negative emotion and absolutist words, and analysis of selfie photos to detect sad facial expressions (Lind et al., 2018; Rohani et al., 2018; Yim et al., 2020). These passive data streams have also shown promise in predicting changes in depressive symptoms over time (De Angel et al., 2022; Dogan et al., 2017).

Complementary use of EMA and actigraphy or mobile sensing offers a comprehensive and reliable approach by linking subjective experiences (e.g., self–reported mood) with objective measures (e.g., sleep, physical activity (Ben-Zeev et al., 2015; Cornet & Holden, 2018))—if adolescents are willing to use them at a sufficiently high level of adherence. Thus, understanding the feasibility and acceptability of these methods among the populations under study is a crucial step for advancing this field. In adult samples, meta-analytic estimates suggest average EMA completion rates of approximately 80% across a range of study designs and populations (Williams et al., 2021; Wrzus & Neubauer, 2023), with wearable device adherence varying more widely across studies (53–87%; Brannon et al., 2016; Tonkin et al., 2023; Zhu et al., 2024). Both tend to decline in longer-duration protocols as well (Tonkin et al., 2023). Whether these benchmarks generalize to community-based adolescents with elevated depressive symptoms remains an open question.

EMA and actigraphy have demonstrated feasibility and acceptability in several studies with suicidal adolescents and those with mood disorders, with EMA completion rates ranging from approximately 73–81% and actigraph wear times averaging 18 or more hours per day (Brannon et al., 2016; Glenn et al., 2022; Kleiman et al., 2019; Zhu et al., 2024). In addition, many of these studies were conducted with higher-acuity clinical samples (e.g., adolescents and young adults discharged from inpatient psychiatric care or emergency departments following a suicide-related crisis) or in non-U.S. contexts, which may limit generalizability to community-based youth in the United States (Czyz et al., 2018; Glenn et al., 2022; Jiang et al., 2023; Zhu et al., 2024). Less is known about the feasibility and acceptability of longer-term, multi-modal protocols specifically among community-recruited adolescents with elevated but subclinical depressive symptoms, a population that is increasingly targeted by digital mental health research yet remains understudied in this context. Beyond population-specific gaps, many EMA studies are also characterized by methodological limitations including using short data collection periods (i.e., one week or less), avoiding school hours, or requiring use of study–provided devices (Baltasar-Tello et al., 2018; Heron et al., 2017; Van Roekel et al., 2019; Wen et al., 2017), making it difficult to draw conclusions about long–term feasibility or acceptability. Despite evidence that adolescents interact less naturally and less frequently with study–provided devices, few studies have allowed use of personal devices, likely due to practical, ethical, and technical challenges (e.g., data privacy, device compatibility, data security) (Cornet & Holden, 2018). Similarly, many passive sensing studies utilize Android devices due to greater access to sensor data despite the predominant use of iOS devices among US adolescents (Cornet & Holden, 2018). These device–related discrepancies may undermine ecological validity and limit sample representativeness. Although using personal devices and sampling during school hours come with logistical challenges (e.g., varying school and caregiver phone–use policies), both are needed for capturing a thorough picture of adolescents’ everyday lives.

### The Present Study

This study examined the feasibility and acceptability of a one–month EMA, wrist actigraphy, and passive mobile sensing protocol for adolescents ages 12–18 years with elevated depressive symptoms. We evaluated (1) protocol engagement through recruitment and retention rates, (2) adherence across modalities by quantifying proportion of EMA surveys completed, average actigraph wear time, and amount and frequency of sensor data transmitted, and (3) acceptability through self–reported satisfaction ratings, qualitative feedback, and barriers to adherence. We also discussed practical challenges of using EMA, actigraphy, and mobile sensing with adolescents experiencing depression, including issues related to participant engagement, device usability, and data collection consistency. In addition, we detailed action steps taken to address these challenges, such as adjusting recruitment strategies and refining protocol instructions, and explored how methodological factors (e.g., survey frequency, actigraph model) influenced adherence and acceptability. We hope to provide valuable information for researchers looking to use these methods by highlighting key considerations for study design and strategies to enhance feasibility and acceptability of similar studies.

## Method

### Participants

Adolescents were eligible to participate in the study if they (1) were 12 to 18 years of age, (2) reported a score of 16 or greater on the Center for Epidemiologic Studies–Depression (CES–D; Radloff, 1977) during phone screening, a threshold that has been used in prior research to indicate elevated levels of depressive symptoms in adolescents (Roberts et al., 1991), (3) owned a compatible smartphone, (4) were fluent in English, and (5) had one caregiver or legal guardian fluent in English or Spanish willing to participate. Exclusion criteria included: (1) changes to current mental health services for depression in the last 4 weeks (e.g., therapy provider, medication dose), to ensure observed symptom changes were not due to recent treatment changes, (2) current PRN pharmacotherapy that could alter mood or related symptoms, (3) past-month acute suicidality (i.e., method, plan, intent), (4) past-year suicide attempt or serious non–suicidal self–injury, (5) history of suicide attempt over a year ago and were not receiving mental health services at time of screening, or (6) history of mania or psychosis.

The total sample included 69 adolescents with a mean age of 15.46 years (*SD* = 1.54) and 66.67% were assigned female at birth. Most (62.32%) identified as heterosexual or straight, with others identifying as gay, lesbian, or queer (7.25%), bisexual (15.94%), undecided or questioning (10.14%), or pansexual (4.35%). Adolescents reported their race as White or Caucasian (42.03%), Black or African American (23.19%), American Indian or Alaska Native (1.45%), Multiracial (7.25%), and Other (15.94%). The remaining 10.14% indicated “I don’t know.” Nearly three–quarters (71.01%) identified as being of Hispanic or Latine ethnicity. The majority (85.51%) were iOS users. Participants were recruited from social media advertisements (e.g., Facebook, Instagram) and community sources in a southeastern region of the United States, including local middle and high schools and a university–based youth mental health clinic.

### Procedure

The data for this study were derived from a larger study examining adolescents’ smartphone use behaviors to identify person–specific “drivers” of depression and to validate passive mobile sensing against EMA and actigraphy. The study was approved by the Institutional Review Board of Florida International University. The full protocol is published elsewhere (Ng et al., 2024). Methods relevant to the current analyses are detailed below.

#### Baseline Assessment

All participants provided electronic written informed consent (adolescents aged 18 years and caregivers) or assent (adolescents aged 12–17 years) using the Research Electronic Data Capture (REDCap) platform. For minors, consent was obtained from both legal guardians when applicable, although only one caregiver participated in the study. Once consented, research staff mailed an ActiGraph watch to participants prior to the baseline visit, which was conducted via Zoom. Participants completed a suicide risk assessment, safety plan, brief interviews, and self–report measures of clinical symptomatology. Adolescents also installed the Effortless Assessment Research System (EARS) app onto their smartphone with guidance from research staff to enable survey notifications and allow necessary permissions for mobile sensors (Lind et al., 2018). Youth were advised to keep EARS running in the background for continuous data collection. Staff also provided an overview of how to use the actigraph, which was worn on their nondominant wrists. To address school device–use policies, a letter was provided for school staff that briefly explained the study and shared the research team’s contact information.

#### Smartphone and Actigraph Data Collection Period

EMA surveys were administered through EARS. Participants received two types of surveys daily: a morning survey about sleep (e.g., bedtime, sleep onset time, wake time, sleep quality) and safety (i.e., suicidal ideation, abuse), and “feelings” surveys throughout each day asking participants to rate 21 items assessing thoughts, moods, and behaviors related to depression (e.g., “felt tired,” “felt happy”) on a scale of 0–100 with higher numbers indicating greater intensity.

Two EMA sampling protocols were used. The initial protocol was completed by 41 participants, lasted 28 days, and was delivered using EARS 1.0. The sleep/safety survey was delivered at 7 AM EST and available until 1 PM. Feelings surveys were delivered five times per weekday between 9 AM to 7 PM EST and six times per weekend day between 9 AM to 9 PM EST, roughly every two hours (i.e., delivered at random times within each 2–hour interval with a buffer of at least 30 min between surveys, thus resulting in response windows varying from 30 to 90 minutes). This protocol was modified using the updated EARS 2.0 based on participant feedback to allow for semi–personalized survey schedules and longer windows for responding to feelings surveys. EARS 2.0 featured greater researcher control over survey design and administration (Lind et al., 2023). The sleep/safety survey was delivered earlier at 5 AM EST to allow completion before the school day. For feelings surveys, participants chose one of three survey schedules starting at 6, 7, or 8 AM EST on weekdays and 8, 9, or 10 AM EST on weekends. Feelings surveys were delivered every three hours at the beginning of each 3–hour interval five times every day, with a response window of 2 hours, thus leaving a buffer of at least 1 hour between surveys. The protocol length was increased to 30 days to account for fewer surveys given on weekends. This modified protocol was given to the 28 most recent participants, of which the first five received 28 days of surveys instead of 30 due to a technical error.

A research–grade actigraph worn on participants’ nondominant wrist was used to collect physical activity and sleep data at consistent intervals. Participants were asked to wear the watch continuously unless near water to minimize risk of damage. The ActiGraph wGT3X–BT was worn by 20 participants, whereas the remaining participants (*n* = 49) wore the ActiGraph GT9X Link. We introduced a new model based on participant feedback regarding preference for a more discreet device. Two features are unique to each model; the wGT3X–BT includes an ambient light sensor, and the GT9X Link features a liquid–crystal display and gyroscope. Both use the same triaxial accelerometer and are designed to achieve comparable results.

Passive mobile data was collected from participants’ smartphones using EARS. Both iOS (*n* = 60) and Android (*n* = 9) users’ data streams included accelerometer, motion (i.e., walking, running, cycling, driving), GPS, selfies, and text typed into the keyboard. For iOS only, battery status (i.e., level, charge), call status, and screen–on time were recorded. Participants were asked to allow permissions for all available sensors as part of enrollment, except for selfies and key input, which were optional. EARS developers made two changes during the course of the study: addition of screen–on time sensor in mid–March 2023 (available for 33 participants), and retirement of selfie sensor in late October 2023 (previously available for 38 participants).

#### Follow–Up Assessment

After the data collection period, participants completed a follow–up visit via Zoom that included self–report measures similar to those given at the baseline visit and instructions for returning the actigraph and uninstalling EARS. Adolescents also reported on protocol acceptability via a self–report measure and qualitative interview. Using a 5–point Likert scale (0 = strongly agree, 4 = strongly disagree), they rated 17 items related to their experience with the actigraph (e.g., it was easy to incorporate wearing the watch into my daily life), EMA surveys (e.g., the number of times I had to answer questions per day was manageable), and mobile sensors (e.g., I felt comfortable allowing the app to record my behaviors for the study). Open–ended questions further explored subjective experiences, including reasons for not wearing the actigraph or completing surveys (see Table S1).

#### Compensation

Participants were compensated $20 per assessment visit and $15 for wearing and returning the actigraph. In the initial EMA protocol, participants earned $0.25 per EMA survey completed plus a $1 daily bonus if they completed all surveys for the day. Participants earned an additional bonus of $5 to $15 for completing 80–95% of total surveys (i.e., 141–168 of 176 possible surveys for 28-day protocol). In the modified protocol, the daily $1 bonus was removed as participants rarely earned it, suggesting that it was not an effective reinforcer. Compensation was increased to $0.50 per survey with a bonus of $5 to $15 for completing 80–90% of total surveys (i.e., 144–162 of 180 possible surveys for 30-day protocol). In total, participants were eligible to receive up to $151 or $160 for high adherence to the 28-day or 30-day study protocol.

#### Participant Contact

Research staff completed a brief check–in with participants on day 1 of the data collection period and monitored their EMA completion and data uploads daily. Positive encouragement and survey progress updates were regularly provided to participants. An initial cohort of participants (*n* = 8) received text messages at the end of each week (i.e., days 7, 14, 21, 28) detailing their compensation earned thus far and a reminder to charge the actigraph. Later participants (*n* = 61) received daily text messages of survey earnings from the previous day, as well as messages on days 7, 14, 21, and 30 with total earnings to date and a reminder to charge the actigraph. Additional contacts were made as needed to address concerns related to EARS or the actigraph (i.e., missing many surveys or data uploads) that arose during daily monitoring.

### Data Analysis Plan

Data analyses were completed using RStudio version 2025.09.0. We computed descriptive statistics for adherence to each data collection method (EMA, actigraph, and mobile sensor) and protocol acceptability. Adherence to EMA was defined as the total number of surveys completed divided by the total number of possible surveys. Adherence to actigraph was estimated based on the total minutes of wear time detected by the actigraph divided by the total number of minutes expected for the one–month data collection period. Adherence to mobile sensing was characterized by the total number of hours with at least 1 observation collected for each data stream. Adherence patterns across data collection methods were examined using Pearson correlations, which were computed among EMA completion rates, actigraph wear time, and mobile sensing data availability. Acceptability was assessed quantitatively by taking the mean ratings on acceptability–related items and qualitatively to identify recurrent themes from participants’ open–ended responses from the follow–up interview. Themes were developed inductively through an iterative review of all open-ended responses by the first author, who organized responses into recurring categories and refined theme labels using a systematic, exploratory approach to summarizing qualitative feedback. In addition, CES-D scores from baseline and follow-up were examined descriptively to assess whether participation was associated with changes in depressive symptoms. To examine methodological factors influencing adherence, we computed correlations within and across modalities and conducted *t*-tests comparing: (a) participation during the academic year versus summer break, (b) weekdays versus weekend days, (c) use of the original app platform (EARS 1.0) with a standardized EMA schedule versus the updated app platform (EARS 2.0) with a semi–personalized EMA schedule, and (d) actigraph model used. Effect sizes are reported as Cohen’s *d* for *t*-tests and Pearson’s *r* for correlations. Following Cohen (1988), *d* values of 0.2, 0.5, and 0.8 represent small, medium, and large effects, respectively, and *r* values of 0.10, 0.30, and 0.50 represent small, medium, and large effects, respectively.

## Results

### Feasibility

**Fig. 1:**
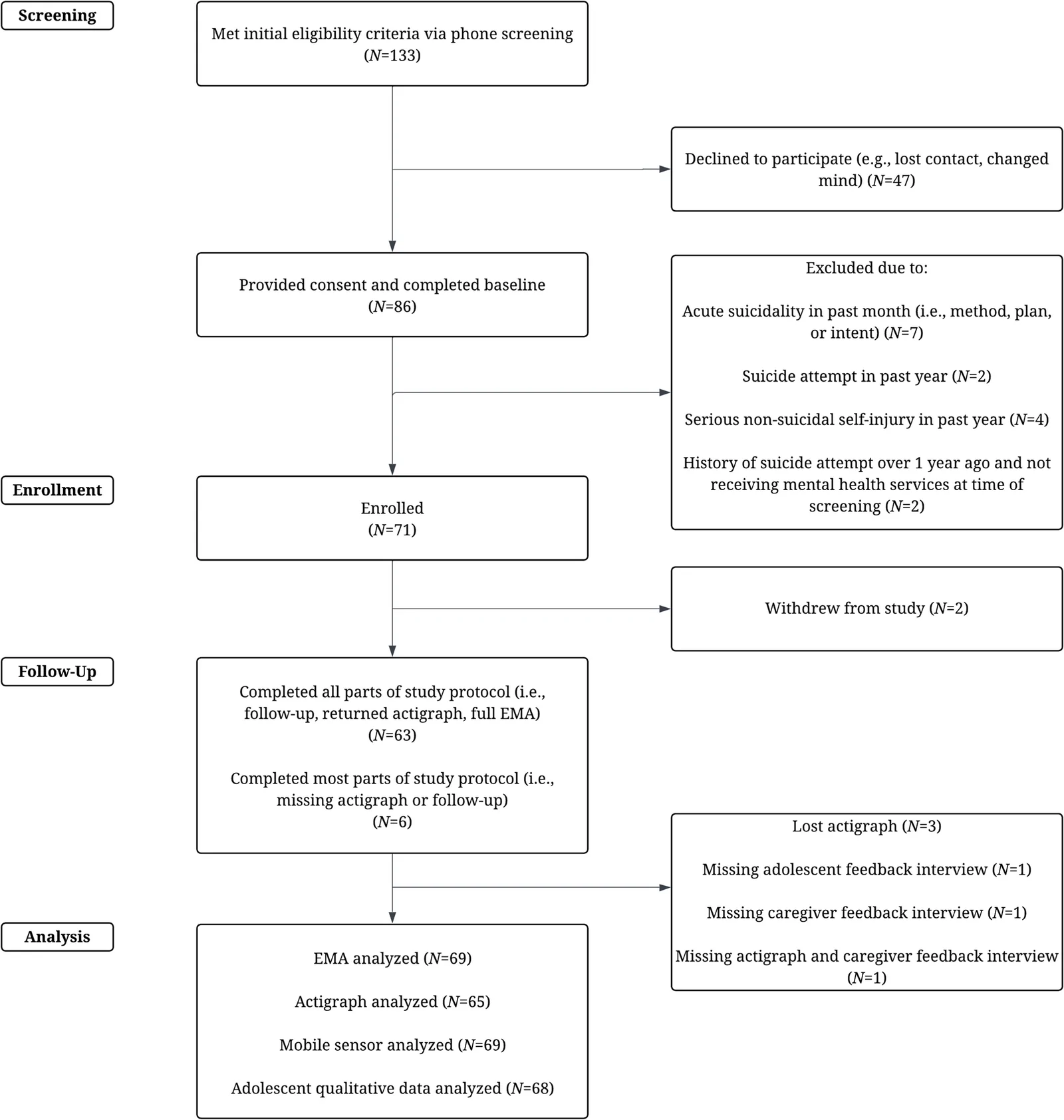
Participant flow diagram

Figure 1 shows the flow of participants from May 2021 through March 2025. During this period, 133 families met initial eligibility criteria at phone screening. Of these, 86 (64.66%) families provided consent and completed a baseline assessment. Seventy–one (82.56%) families were eligible at baseline and completed full enrollment procedures, including downloading EARS and wearing the actigraph. Two participants withdrew from the study before completing the full study protocol. Four participants did not have actigraph data available due to lost devices. One participant did not have follow–up data available. The total analytic sample included 69 for EMA, 65 for actigraphy, 69 for mobile sensing, and 68 for qualitative feedback. As a secondary observation to the aims of this study, CES-D depressive scores did not significantly change from baseline (*M* = 23.65, *SD* = 9.76) to follow-up (*M* = 22.09, *SD* = 9.48; *t*(67) = −1.38, *p *= 0.172, *d *= −0.17), suggesting that participation was not associated with meaningful worsening of mood.

### EMA Adherence

**Fig. 2:**
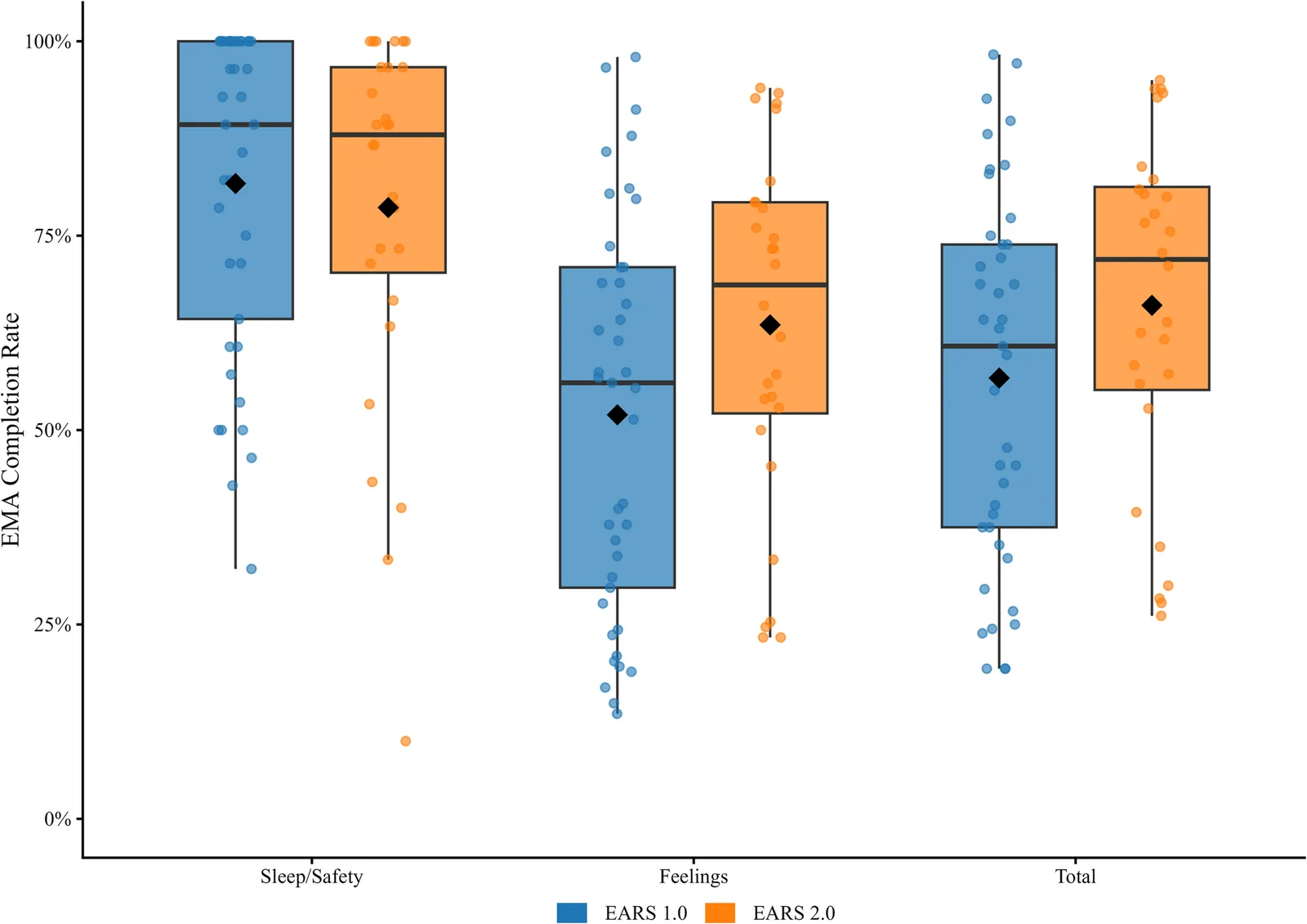
Individual and sample mean EMA completion rates by survey type and EARS version. Each point represents an individual participant’s completion rate. Boxes indicate the interquartile range, horizontal lines indicate the median, whiskers extend to 1.5 times the interquartile range, and black diamonds indicate the group mean. EARS 1.0 means: sleep/safety = 81.71%, feelings = 51.96%, total = 56.69%. EARS 2.0 means: sleep/safety = 78.61%, feelings = 63.53%, total = 66.04%

During the one–month data collection period, the 69 participants completed 60.49% of all surveys (*M* = 106.94, *SD* = 41.96). On average, participants completed 22.83 sleep/safety surveys (*SD* = 6.40) and 83.90 feelings surveys (*SD* = 36.76). See Fig. 2. About one–fourth of participants (*n* = 18; 26.09%) completed at least 75% of all surveys, and about two–thirds (*n* = 45; 65.22%) completed at least 50% of all surveys.

There was no significant difference in total survey completion rates found between participants who completed the study during the academic year compared to summer break (*t*(14.08) = –0.29, *p = *0.78, *d *= –0.09), as well as participants who did and did not receive daily text messages regarding survey earnings (*t*(9.93) = –1.23, *p = *0.25, *d *= –0.40). Participants who used EARS 2.0 completed more surveys overall (*M* = 117.43, *SD* = 39.95) compared to those who used EARS 1.0 (*M* = 99.78, *SD* = 42.25), but this difference did not reach statistical significance (*t*(60.93) = –1.66, *p = *0.10, *d *= –0.40). Similarly, EARS 2.0 participants completed more feelings surveys overall (*M* = 94.14, *SD* = 33.93) than EARS 1.0 participants (*M* = 76.90, *SD* = 37.36), with a marginal difference observed (*t*(62.06) = –1.99, *p = *0.05, *d *= –0.48). There was also no significant difference in weekday and weekend total completion rates (*t*(68) = –0.25, *p = *0.80, *d *= –0.03).

### Wrist Actigraphy Adherence

**Fig. 3:**
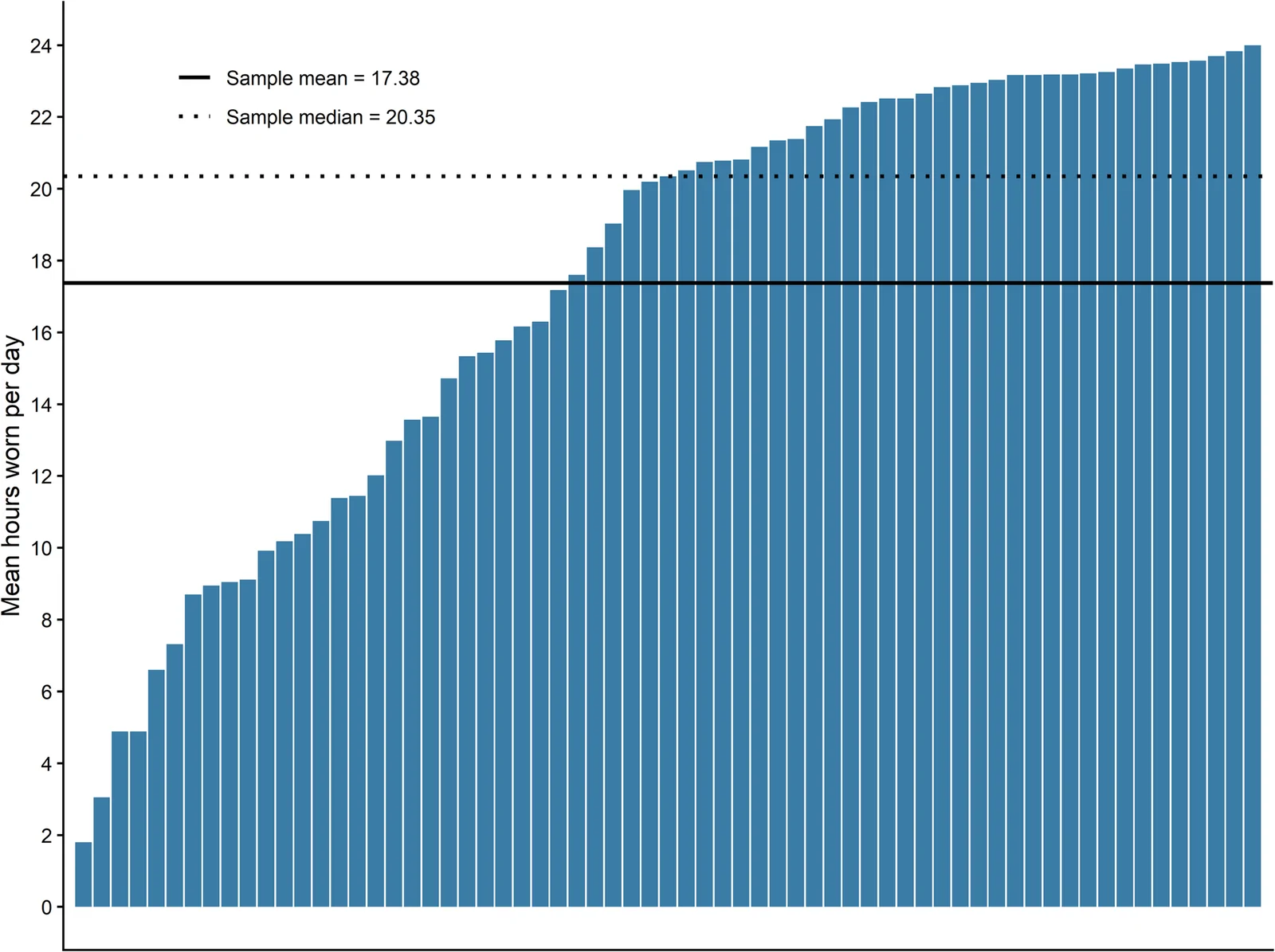
Sample mean daily actigraph wear time in hours (*n* = 65). Each bar indicates the mean number of hours worn by each participant across the data collection period

During the one–month data collection period, 65 participants wore the actigraph for a mean of 72.40% of the time (about 17 hours per day; see Fig. 3). More than half (*n* = 37; 56.92%) wore the device at least 80% of the time.

Actigraph wear time did not significantly differ between participants who wore the wGT3X-BT model compared to those who wore the GT9X Link (*t*(31.07) = 0.92, *p = *0.36, *d* = 0.26) or between participants who completed the study during the academic year compared to summer break (*t*(16.42) = 0.19, *p = *0.85, *d* = 0.05). There was a significant difference between wear time on weekdays compared to weekends (*t*(64) = 3.70, *p < *0.001, *d* = 0.46). Average wear time was higher on weekdays (*M* = 74.10%, *SD* = 26.02%) compared to weekends (*M* = 68.51%, *SD* = 28.82%).

### Mobile Sensing Data Availability

**Fig. 4:**
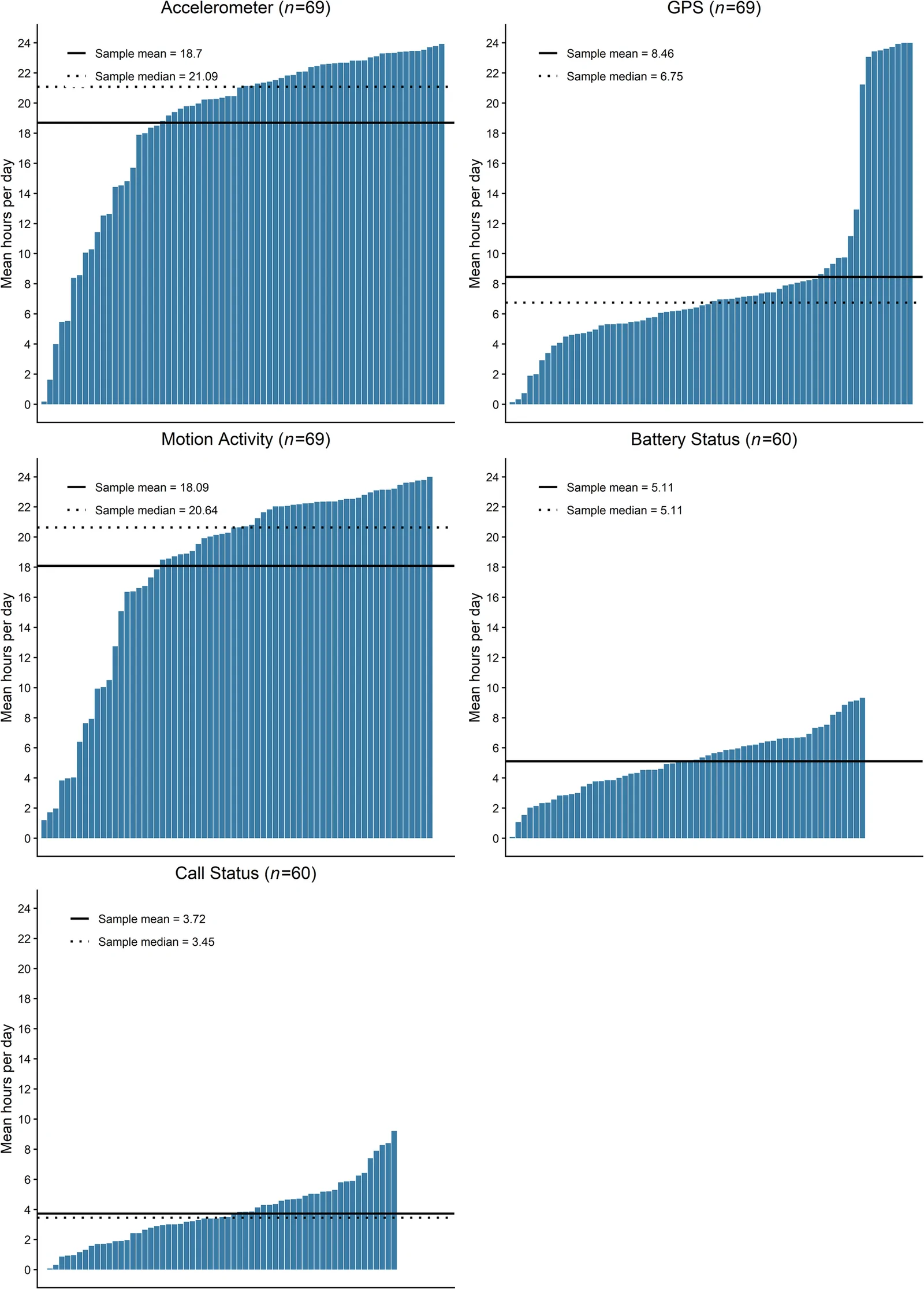
Sample mean number of hours with ≥ 1 observation for each sensor for each participant. Accelerometer and motion activity sensors for four participants (iOS13, iOS16, iOS44, iOS55) were affected by technical issues that caused data loss

**Fig. 5:**
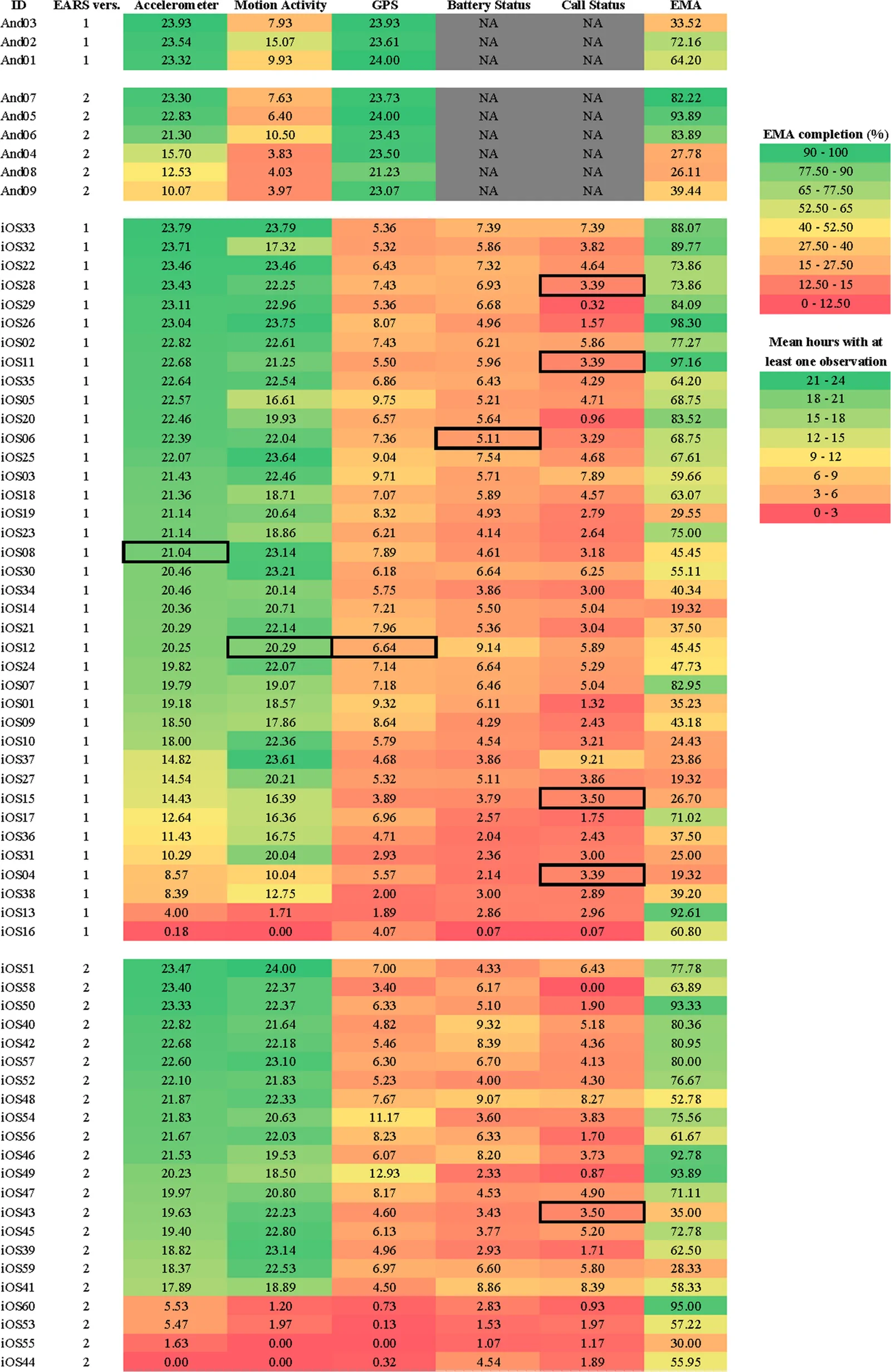
Heat map of mean number of hours with ≥ 1 observation by sensor and participant; black boxes mark median number of hours per sensor; IDs with “iOS” represent iOS users and “And” represent Android users; accelerometer and motion activity sensors for four participants (iOS13, iOS16, iOS44, iOS55) were affected by technical issues that caused data loss

Data for seven mobile sensors (accelerometer, battery status, call status, GPS, motion activity, key input, and selfie) were available for the 69 participants. Battery and call status sensors were available only for iOS users (*n* = 60, 86.96%). Sample mean number of hours with at least one observation for each sensor can be seen in Figs. 4 and 5. Notable sensor differences between the iOS and Android versions of EARS are detailed below.

The key input sensor was a required sensor for EARS 1.0 participants (*n* = 41) and optional for EARS 2.0 (*n* = 28). We made the decision to change the requirement based on feedback regarding privacy concerns related to keyloggers and usability (i.e., individual preference for native keyboard over EARS keyboard due to ease of use). Of the 28 EARS 2.0 participants, 5 (17.86%) opted to enable the key input sensor. Key input data were captured when the EARS custom keyboard was in use in non–secure fields (i.e., no data from secure text fields) and were available for 46 participants.

Of 38 participants with access to the selfie sensor, 33 (86.84%) opted to enable it. Photos taken and saved to the device’s camera roll with the participant’s face (identified based on reference photo taken during baseline) were captured, excluding photos with multiple individuals or without the participant’s face. The selfie sensor collected a mean of 3 photos with a range of 0–24 photos across participants. Twelve participants (35.29%) had at least 1 photo collected.

The accelerometer, motion activity, and GPS sensors differed functionally between iOS (*n* = 60) and Android (*n* = 9) users. For accelerometer, data were sampled continuously for both operating systems. However, Android devices had a minimum threshold of acceleration needed to trigger a data capture while iOS devices did not. Motion data type (e.g., stationary, walking, running, cycling, automotive) were classified using OS-specific algorithms. For GPS, iOS devices sampled data continuously and captured locations change over 100 meters. Android devices sampled latitude and longitude coordinates every 15 min with no threshold for data capture. The accelerometer sensor collected a mean of 18.43 hours with at least one observation (*SD* = 6.25). Similarly, the motion activity sensor obtained a mean of 17.30 hours (*SD* = 7.31). In contrast, the GPS sensor recorded a mean of 8.34 hours (*SD* = 6.33).

Battery and call status sensors captured discrete events (e.g., charging phone, making or receiving a phone call). On average, participants had fewer than 10 hours with at least one observation for battery status (*M* = 5.11, *SD* = 5.11) and call status (*M* = 3.72, *SD* = 3.45).

### Adherence Patterns Within and Between Modalities

To examine adherence patterns across data collection methods, Pearson correlations were computed between EMA completion rates, actigraph wear time, and availability for each mobile sensor data stream (see Table S2). Significant positive correlations were observed within modalities. EMA completion rate was strongly correlated between survey types (i.e., sleep/safety and feelings surveys; *r* = 0.78 to 1, median *r* = 0.83, *p < *0.001). Accelerometer sensor availability was weakly to strongly correlated with other sensor streams (*r* = 0.25 to 0.71, median *r* = 0.46, *p < *0.05). Battery sensor availability was most strongly correlated with accelerometer, call, and motion sensors (*r* = 0.71, 0.50, and 0.49 respectively, *p < *0.001), and was not significantly correlated with GPS (*r* = 0.25). Call sensor availability showed weak to moderate correlations with other sensors (*r* = 0.31 to 0.50, median *r* = 0.31, *p < *0.05), except for GPS (*r* = 0.16), which was not significant. GPS sensor availability was weakly correlated with accelerometer (*r =* 0.25, *p < *0.05) but strongly negatively correlated with motion (*r = *–0.47, *p < *0.001) sensors and not significantly correlated with other sensors. Motion sensor availability showed weak to strong correlations with all other sensors (*r *= –0.47 to 0.60, median *r* = 0.40, *p < *0.05).

Between–modality correlations were lower on average. Total EMA completion rate was weakly to moderately correlated with actigraph wear time (*r* = 0.28, *p < *0.05) and accelerometer (*r =* 0.37, *p < *0.01) sensors. Correlations between total EMA completion rate and other mobile sensors were not significant (*r *= –0.11 to 0.23, median *r* = 0.02). Actigraph wear time was moderately correlated with accelerometer (*r* = 0.40, *p < *0.01) and battery sensors (*r* = 0.30, *p < *0.05) but not with other sensors (*r* = 0.03 to 0.20, median *r* = 0.04).

### Protocol Acceptability

All but one (98.55%) adolescent completed the feedback interview at follow-up; one adolescent felt that the compensation for the follow–up visit was inadequate. The majority indicated that the protocol was acceptable and reported a generally positive experience wearing the actigraph, completing EMA surveys, and using EARS (see Table 1). Common barriers to adherence included temporary device removal and limited phone access at school or during activities when actigraph or phone use were not recommended (see Table 2).

**Table 1:** Participant perception and acceptability of protocol (*n* = 68)

Protocol Component	Item	EARS 1.0 *M* (*SD*)	EARS 2.0 *M* (*SD*)	Total *M* (*SD*)	Total *N* (% Endorsed Agree or Strongly Agree)
Wrist actigraphy	I enjoyed using the actigraph (watch)	2.67 (0.83)	2.79 (0.96)	2.72 (0.88)	47 (69.12)
	It was easy to incorporate wearing the watch into my daily life	3.15 (0.92)	3.14 (1.04)	3.15 (0.97)	58 (85.29)
	Using the watch disrupted my daily activities	0.98 (0.95)	1.00 (0.98)	0.99 (0.95)	7 (10.29)
EMA	I enjoyed answering the survey questions on the app	2.70 (0.88)	2.68 (1.02)	2.69 (0.93)	46 (67.65)
	It was easy to answer the questions on the app	3.20 (0.61)	3.36 (0.91)	3.26 (0.75)	61 (89.71)
	The number of days I had to answer questions was manageable (i.e., 28 days was not too long)	3.17 (0.59)	3.11 (0.63)	3.15 (0.61)	60 (88.24)
	The number of times I had to answer questions per day was manageable (i.e., 6–7 times per day was not too many)	2.58 (0.90)	2.82 (0.94)	2.68 (0.92)	48 (70.59)
	The number of questions I had to answer each time was manageable	2.72 (0.64)	3.04 (0.84)	2.85 (0.74)	54 (79.41)
	The timing of the questions worked with my schedule-I had enough time to answer them before they went away	2.05 (0.96)	2.32 (1.09)	2.16 (1.02)	28 (41.18)
	Answering questions on the app disrupted my daily activities	1.20 (0.82)	1.14 (0.85)	1.18 (0.83)	5 (7.35)
Mobile sensing	I was worried about my own privacy while having the app on my phone	1.62 (1.17)	1.43 (1.14)	1.54 (1.15)	16 (23.53)
	I felt comfortable allowing the app to record my behaviors for the study	3.02 (0.66)	3.07 (0.60)	3.04 (0.63)	62 (91.18)
Overall	I would participate in a study like this again in the future	3.12 (0.65)	3.46 (0.64)	3.26 (0.66)	62 (91.18)
	I would participate in a study like this again if my doctor or counselor told me that it can help me to learn more about my feelings and figure out ways to feel better	3.38 (0.49)	3.32 (0.94)	3.35 (0.71)	66 (97.06)
	I would recommend participating in a study like this to my good friends	3.38 (0.59)	3.14 (0.71)	3.28 (0.64)	63 (92.65)

Items were rescaled such that higher scores reflect greater agreement (i.e., 0 = strongly disagree, 4 = strongly agree)

**Table 2:** Common participant reported barriers to protocol adherence

Protocol Component	Reason	*N* (% Endorsed)
Wearing actigraph	Forgetfulness after removing device for event or activity (e.g., after showering, going to the beach)	28 (41.18)
	Intentional removal of device for an event or activity (e.g., water sports, showering, sleeping)	18 (26.47)
	Hardware–related issues (e.g., watch strap broke, replacement needed)	5 (7.35)
	Removed due to skin irritation	5 (7.35)
Completing EMA surveys	Limited phone access at school	25 (36.76)
	Not near phone during survey window (e.g., sleeping, participating in an activity)	22 (32.35)
	Survey schedule did not align well with personal schedule (e.g., travel, “too busy”)	18 (26.47)

## Discussion

The current study examined feasibility and acceptability of a one–month multimodal assessment protocol for adolescents with elevated depressive symptoms. To the best of our knowledge, our study is one of the first to use a combined approach of EMA, actigraphy, and mobile sensing with this population. More than 85% of enrolled participants completed all parts of the study, despite the fairly long protocol and intense EMA sampling schedule. Moreover, participants’ adherence rates to EMA, actigraphy, and mobile sensing were within the range with those found in previous studies (50–90%) (Heron et al., 2017; Wen et al., 2017). The high completion rate suggests that adolescents with depression and their caregivers are willing to engage with these data collection methods.

### EMA

Completion rates for the once–daily sleep/safety surveys were high (~ 80%), whereas the more frequent feelings surveys had lower response rates (~ 50–60+%). We attempted to improve response rates with schedule changes. In EARS 1.0, prompts were delivered on a variable–interval schedule every two hours with response windows varying from 30 to 90 minutes. In EARS 2.0, prompts were delivered on a fixed–interval schedule every three hours with consistent 120–minute response windows and semi–personalized daily start and end times to better match participants’ everyday routines. On average, EARS 2.0 participants completed more feelings surveys (94.14) compared to EARS 1.0 participants (76.90), suggesting that scheduling adjustments can lead to modest improvements in adherence. Although this difference was only marginally significant in our sample, personalizing schedules may still be a worthwhile consideration for maximizing response rates. These completion rates are lower than meta-analytic averages reported in adult EMA literature (~ 80%; Williams et al., 2021; Wrzus & Neubauer, 2023), though they are more comparable to rates observed in longer-duration adult protocols (Tonkin et al., 2023). This suggests that protocol length, sampling frequency, and developmental factors may each contribute to adherence patterns in adolescent samples. For greater impact on adherence, researchers may need to consider more substantial changes in study design such as reduced frequency or number of items, reduced study duration, sampling bursts during times when unlimited access to the research devices is expected, or more extensive gamification (Heron et al., 2017).

Lower completion of feelings surveys may reflect greater cognitive and emotional burden associated with identifying affective states, as well as the discomfort of engaging with negative emotions multiple times per day. The sleep/safety surveys had longer response windows (6 + hours) and lower repetitiveness (daily and first survey of the morning) that likely contributed to higher rates of completion. Nonetheless, most participants found the protocol manageable in terms of overall duration (*n* = 60, 88.24% reported Agree or Strongly Agree), number of surveys per day (*n* = 48, 70.59% reported Agree or Strongly Agree), and number of items per survey *(n* = 54, 79.41% reported Agree or Strongly Agree). Although these response rates suffice for analyses that aggregate responses across days or weeks, they pose greater limitations for lagged or dynamic prediction models, where a single missing datapoint results in two missing lagged datapoints (before and after the missing datapoint).

### Wrist Actigraphy

Participants averaged about 17 hours of daily actigraph wear, with more than half (*n* = 37; 56.92%) wearing the device at least 75% of the time. Most participants reported that they enjoyed using the actigraph (*n* = 47, 69.12% reported Agree or Strongly Agree) and found it easy to incorporate into their daily lives (*n* = 58, 85.29% reported Agree or Strongly Agree). The most common reasons for non–wear were temporarily removing the device for specific activities (e.g., sports, showering; *n* = 18, 26.47%) and forgetting to put it back on after an event or activity (e.g., going to the beach, post–shower; *n* = 28, 41.18%). Given the coastal location and subtropical climate of the study location, participation in beach and water activities was likely higher in our participant sample compared to studies in other locations.

Some participants reported discomfort with wearing the device due to its aesthetics, describing it as “bulky” and less attractive compared to commercially available wearables. However, research–grade devices have demonstrated greater validity and reliability for measuring sleep and physical activity in children and adolescents compared to commercial devices (Schmidt et al., 2023). In response to participants’ concerns, we transitioned from the larger red ActiGraph wGT3X-BT to its sleeker black counterpart, the ActiGraph GT9X Link. Interestingly, we received comparable comments about both models with no significant difference in wear time, suggesting that the two were similarly acceptable to participants on average. Actigraphy wear time in the current sample (~ 17 hours/day) is particularly encouraging given that adolescents have been shown to exhibit greater wear fatigue—or day-to-day decline in device wear—compared to adults in large population-based samples (Lamunion et al., 2024). Our participants maintained relatively consistent wear over the one-month protocol period, suggesting that a combination of regular monitoring, timely outreach, and device problem-solving may help offset typical adolescent wear fatigue. Of note, we also experienced several hardware–related issues, including the device holster or strap breaking during use and data download failures. Although data were not lost, devices needed to be sent back to the manufacturer for manual extraction, which reduced device availability and slowed data collection. These challenges highlight the importance of balancing research–grade accuracy with participant preferences for aesthetics and wearability.

### Mobile Sensing

Sensor adherence appeared to depend on the thresholds for triggering data capture, which in turn varied as a function of device operating system. Sensors that sampled data continuously or had low thresholds for data capture, such as accelerometer and motion activity, recorded data for an average of over 17 hours per day. On the other hand, sensors with higher thresholds, such as GPS, or those that captured only discrete events (e.g., charging phone, making or receiving a phone call), recorded data for less than 10 hours per day on average. The selfie sensor was an optional feature available to a subset of participants (*n* = 38); of these, most (87%) opted to enable it. The high opt–in rate suggests that many participants were comfortable having photos of themselves collected and analyzed, although the resulting photo data were relatively sparse. Only 12 participants (35%) had at least one photo captured, and the number of photos collected from each participant varied widely (0–24; *M* = 3.09, *SD* = 5.59). This limited yield constrains the feasibility of analyses requiring sufficient photo data (e.g., facial expression detection for mood inference). In contrast, only one–fourth of the participants who were given a choice opted in to turn on the key input sensor, and several reported usability issues (e.g., disruptions or pauses in typing), which led them to disable it despite initially opting in.

Mobile sensing raises important privacy considerations, given the increased level of risk associated with and often sensitive nature of continuous smartphone data collection. Although most participants felt comfortable allowing the EARS app to record their behaviors (91.18% Agree or Strongly Agree), about a quarter expressed concern about privacy (23.53% Agree or Strongly Agree), and several families declined participation during or after phone screening for these reasons. To address these concerns, we implemented several safeguards, including transparent communication about data security, clear explanations of what data would be collected, and the option to withdraw from the study at any time. Our IRB also required informed consent from all legal guardians (even though only one was asked to participate in the study) for participants who were minors, noting concerns about the perceived invasiveness of data collection. We emphasized that the EARS app follows best practices for data protection (Lind et al., 2018, 2023). Even so, there may be selection bias toward participants who felt less concerned about privacy of smartphone data. As passive sensing research continues to advance, ethical questions remain a crucial part of the discussion on the utility of these tools (Bryan et al., 2024).

### Patterns of Adherence Within and Between Modalities

We found moderate to strong within–modality correlations and markedly variable between–modality correlations. This pattern suggests that participant-level factors, such as motivation or conscientiousness, play a significant role in adherence across different data collection methods, independent of modality-specific barriers. That is, some participants consistently had more data available within and between modalities compared to other participants, data availability depended in part on the specific type of sensor or survey. In addition, some sensor data (e.g., accelerometer) were less likely to be influenced by user behavior or motivation compared to others (e.g., EMA surveys), indicating that modality–specific challenges played a role in adherence. Participants may face EMA survey fatigue or difficulties with device permissions specific to mobile sensors. For actigraph, wearability is a unique factor for engagement. These patterns emphasize the need to address both participant–level factors and modality–specific challenges to adherence. Future work should examine individual-difference characteristics, such as age, symptom severity, and phone use habits, as predictors of adherence variability, which may inform targeted strategies for improving engagement in future studies.

### Lessons Learned

1. User–centered designs improve adherence. Prior literature emphasizes a strong need for thoughtful study design to optimize adherence, particularly for adolescents. Strategies such as user–friendly interfaces, gamification features, and personalized schedules help minimize fatigue (Heron et al., 2017). In our study, tailoring EMA schedules to better align with adolescents’ daily lives likely contributed to the increased response rates we saw among EARS 2.0 participants.
2. Device choice affects data collection. EARS is one of few research–grade EMA and passive sensing apps available on both iOS and Android at the time of this study. Most passive sensing studies to date have used Android devices (Cornet & Holden, 2018). This is partly because iOS requires additional steps for users to grant permissions to third–party apps. These restrictions make it easier for apps like EARS to function fully on Android devices. Furthermore, operating system differences can affect the frequency and quantity of data captured, which may influence both data quality and the types of analyses that can be conducted. Consideration of device preferences among adolescents is also important for ensuring representative sampling, given that over 88% of US adolescents use iOS devices (Piper Sandler, n.d.).
3. Regular monitoring of compliance is important. Frequent monitoring of protocol adherence allows researchers to quickly identify issues such as device malfunctions, low survey response rates, or data transmission problems. We checked the EARS dashboard daily to catch and address problems early (e.g., app closed, missing many surveys, sensors not uploading). Timely outreach prevents extended gaps in data collection and allows for tailored support (e.g., troubleshooting technical issues, sending reminders to charge watch), which can reduce participant burden and improve overall data completeness. Optimal monitoring frequency may vary for each project depending on several factors, including staff availability, associated population risks, and how resilient the analyses of choice are to missing data.
4. Data collection during school hours is feasible with appropriate planning. Although we anticipated challenges due to school phone–use policies, few participants reported difficulties using their phones at school. Most were able to complete surveys during breaks or between classes. We found no significant differences between participants who completed the study during the academic year versus summer breaks. Flexible response windows and semi–personalized scheduling helped minimize disruptions to their daily routines. Additionally, many participants were recruited from local schools with which our lab had built strong relationships. Offering mental health psychoeducation presentations and resource materials helped foster trust and support from school administrators, which likely facilitated participation.
5. Addressing privacy concerns is essential for participant trust and retention. Privacy concerns can significantly influence recruitment and enrollment. Clear communication about how data is collected, used, and protected is crucial for building trust. Providing transparency, secure data storage, and participant control over their data (e.g., option to withdraw) are key ways to prioritize privacy protections and ethical integrity (Jacobson et al., 2020).

### Limitations

First, findings should be interpreted with the caveat that data were collected across multiple study periods that differed in several concurrent ways, including device type (60 iOS users and 9 Android users), app platform and EMA sampling schedule (41 using EARS 1.0 and 28 using 2.0), compensation structure, and app functionality due to the app developers, iOS, or Android retiring or introducing sensors and algorithms. Because these changes occurred simultaneously, observed differences in adherence between protocol versions—such as the higher feelings survey completion rates among EARS 2.0 participants— should be interpreted cautiously and cannot be attributed to any single modification (e.g., semi-personalized scheduling) independent of other concurrent changes (e.g., increased per-survey compensation, extended response windows). The sample was also geographically limited to Florida (moreover, primarily South Florida) and predominantly female, White, and Hispanic or Latine. Future research should replicate these findings in larger, more diverse samples to assess potential moderators of adherence, such as phone use habits, symptom severity, and cultural factors. Second, although sampling during school hours was a notable strength, further research is needed to evaluate long–term feasibility. Sustaining engagement over extended periods—especially across changing school schedules and extracurriculars—will be essential for applying these methods in clinical or naturalistic settings. Third, although we examined adherence within and between modalities, future work should build on this by exploring dynamic or predictive relationships (e.g., whether EMA compliance forecasts actigraph wear time) and time–varying predictors of adherence (e.g., daily changes in daily mood or sleep). Although our EMA completion rates are sufficient for analyses that aggregate responses across days, they may limit more sophisticated models (e.g., lagged analyses or real–time predictions), which require high–frequency, low–missingness data (Langener et al., 2024). Future work should also focus on establishing the minimum data density required for JITAI algorithms to be effective. Fourth, device–related challenges remain a limitation. Data flow was disrupted by iOS–Android sensor differences and occasional hardware issues (e.g., strap breakage, data download errors). Fifth, sensor–specific challenges complicate interpretations of adherence. For sensors that capture discrete events (e.g., battery, call), data availability reflects the frequency of those behaviors rather than participant compliance or sensors’ data capture capabilities. Limited data from these streams does not indicate non–adherence but rather lower event occurrence. Continued improvements in cross–platform functionality, user–centered device design, and real–time monitoring tools are essential for supporting both data quality and participant experience.

### Future Directions

Multimodal methods hold promise not only for assessment but also for intervention. Continuous tracking of behaviors and physiological markers—such as sleep, activity, and communication—can detect moment–to–moment mood shifts and early signs of symptom worsening (Cao et al., 2020; Sequeira et al., 2020). This is especially valuable for adolescents with depression, who may struggle with self–awareness or recalling mood patterns, limiting treatment effectiveness (Ben-Zeev et al., 2015). Objective data from smartphones and wearables can complement self–monitoring and inform personalized treatment plans. For instance, if sedentary behavior is linked to worsening mood, clinicians can help adolescents set targeted activity goals. These insights also lay the groundwork for just–in–time adaptive interventions that offer timely, tailored support—such as prompting movement or coping strategies in response to real–time indicators like prolonged inactivity or poor sleep (Nahum-Shani et al., 2018). An important open question for the field is whether repeated mood prompting over extended periods may itself influence participants’ affect. Although pre-post depressive symptom data from this sample did not suggest clinically meaningful worsening of depressive symptoms over the assessment period, future research should examine EMA reactivity more rigorously using within-person designs to directly evaluate whether and for whom intensive mood sampling may have iatrogenic effects.

## Conclusion

Together, our findings highlight both the promise and complexity of using EMA, actigraphy, and passive sensing to study adolescent depression. With thoughtful design, proactive planning, and ethical care, intensive data collection methods can be successfully implemented in real–world adolescent contexts. Importantly, our experiences highlight the need to balance methodological rigor with participant–centered approaches—particularly when engaging youth. These insights may inform future research aiming to harness smartphones and wearables in clinically meaningful and scientifically grounded ways.

## Supplementary Information

Below is the link to the electronic supplementary material.

Supplementary Material 1 (DOCX 45.8 KB)

## Data Availability

The datasets generated and analyzed for this study are available from the corresponding author on reasonable request. Anonymized, raw study data are accessible in the National Institute of Mental Health Data Archive with identifier 10.15154/1a1n-3t90.
