# Supplementary material for: Feasibility and Acceptability of a Smartphone and Wearable Assessment Protocol for Adolescents with Depression
Source: Res Child Adolesc Psychopathol. 2026 Jun 18;54(4):78. doi: 10.1007/s10802-026-01464-9 (PMC13279485; doi:10.1007/s10802-026-01464-9)
Supplement: Supplementary file 1 — Supplementary Material 1 (DOCX 45.8 KB) [file 10802_2026_1464_MOESM1_ESM.docx]

**Table S1.** Adolescent feedback interview questions administered at follow–up.

| **Protocol Component** | **Item** |
| --- | --- |
| Actigraph | What did you like about using the actigraph watch? |
|  | What did you dislike about using the actigraph watch? |
|  | If you missed some days of wearing the watch, what got in the way of you wearing it? |
|  | Did you have any worries or concerns about the watch before you started wearing it or while wearing it? |
|  | Was wearing the watch what you expected?  If no, how was it different? |
|  | Did wearing the watch have any effect on your everyday life? If yes, in what way(s)? |
|  | Did you charge the watch?   - If yes, how many times, roughly when? Were there any issues with charging it? - If no, why not? |
|  |  |
| EARS | What did you like about the app? |
|  | What did you dislike about the app? |
|  | If you missed some surveys, what got in the way of you completing them? |
|  | What might have helped or motivated you to complete surveys?   - Did you find it encouraging to receive texts about your progress and how much you earned each day? |
|  | Did you have any worries or concerns about the app before you started using it or while you were using it? |
|  | What concerns did you have about using the EARS keyboard, if any?   - For iOS users: Were you as likely to use the EARS app as the default Apple keyboard? Why or why not? |
|  | Did using the app have any effect on your everyday life?   - If yes, in what way(s)? |
|  | Would you make any changes to the app?   - If yes, what change(s) (e.g., interface design, keyboard, timing of surveys, tracking of survey progress, EXP points)? |
|  | What features would you add to the app? |

**Table S2.** Correlation matrix of EMA completion rates, actigraph wear time, and mobile sensing data availability.

| Variable | 1 | 2 | 3 | 4 | 5 | 6 | 7 | 8 |
| --- | --- | --- | --- | --- | --- | --- | --- | --- |
| 1. actigraph |  |  |  |  |  |  |  |  |
| 1. feelingsEMA | 0.25* |  |  |  |  |  |  |  |
| 1. sleep_safetyEMA | 0.41*** | 0.78*** |  |  |  |  |  |  |
| 1. totalEMA | 0.28* | 1*** | 0.83*** |  |  |  |  |  |
| 1. accelerometer | 0.40** | 0.34** | 0.44*** | 0.37** |  |  |  |  |
| 1. gps | 0.03 | 0.01 | –0.09 | –0.01 | 0.25* |  |  |  |
| 1. battery | 0.30* | 0.22 | 0.30* | 0.23 | 0.71*** | 0.25 |  |  |
| 1. call | 0.04 | –0.11 | –0.08 | –0.11 | 0.31* | 0.16 | 0.50*** |  |
| 1. motion | 0.20 | 0.02 | 0.23 | 0.05 | 0.60*** | –0.47*** | 0.49*** | 0.31* |

*Note.* *=*p<*.05, **=*p<*.01, ***=*p<*.001.
